# Task Shifting Provision of Contraceptive Implants to Community Health Extension Workers: Results of Operations Research in Northern Nigeria

**DOI:** 10.9745/GHSP-D-15-00129

**Published:** 2015-09-10

**Authors:** Zulfiya Charyeva, Olugbenga Oguntunde, Nosa Orobaton, Emmanuel Otolorin, Fatima Inuwa, Olubisi Alalade, Dele Abegunde, Saba’atu Danladi

**Affiliations:** ^a^​ Palladium, Washington, DC, USA; ^b^​ Targeted States High Impact Project (TSHIP), Bauchi, Nigeria; ^c^​ JSI Research & Training Institute, Arlington, VA, USA; ^d^​ Jhpiego – an affiliate of Johns Hopkins University, Abuja, Nigeria

## Abstract

**Background:**

Contraceptive use remains low in Nigeria, with only 11% of women reporting use of any modern method. Access to long-acting reversible contraceptives (LARCs) is constrained by a severe shortage of human resources. To assess feasibility of task shifting provision of implants, we trained community health extension workers (CHEWs) to insert and remove contraceptive implants in rural communities of Bauchi and Sokoto states in northern Nigeria.

**Methods:**

We conducted 2- to 3-week training sessions for 166 selected CHEWs from 82 facilities in Sokoto state (September 2013) and 84 health facilities in Bauchi state (December 2013). To assess feasibility of the task shifting approach, we conducted operations research using a pretest–posttest design using multiple sources of information, including surveys with 151 trained CHEWs (9% were lost to follow-up) and with 150 family planning clients; facility observations using supply checklists (N = 149); direct observation of counseling provided by CHEWs (N = 144) and of their clinical (N = 113) skills; as well as a review of service statistics (N = 151 health facilities). The endline assessment was conducted 6 months after the training in each state.

**Results:**

CHEWs inserted a total of 3,588 implants in 151 health facilities over a period of 6 months, generating 10,088 couple-years of protection (CYP). After practicing on anatomic arm models, most CHEWs achieved competency in implant insertions after insertions with 4–5 actual clients. Clinical observations revealed that CHEWs performed implant insertion tasks correctly 90% of the time or more for nearly all checklist items. The amount of information that CHEWs provided clients increased between baseline and endline, and over 95% of surveyed clients reported being satisfied with CHEWs’ services in both surveys. The study found that supervisors not only observed and corrected insertion skills, as needed, during supervisory visits but also encouraged CHEWs to conduct more community mobilization to generate client demand, thereby promoting access to quality services. CHEWs identified a lack of demand in the communities as the major barrier for providing services.

**Conclusion:**

With adequate training and supportive supervision, CHEWs in northern Nigeria can provide high-quality implant insertion services. If more CHEWs are trained to provide implants and greater community outreach is conducted to generate demand, uptake of LARCs in Nigeria may increase.

## Abstract (precis)

With training and supportive supervision, male and female Community Health Extension Workers (CHEWs) in Nigeria safely and effectively provided contraceptive implants, and virtually all clients said they were satisfied. Most CHEWs achieved competency after 5 client insertions. However, the CHEWs provided only an average of 4 insertions per health facility per month. Realizing the true potential of providing implants calls for a context with dedicated providers and robust outreach.

## INTRODUCTION

Among family planning methods, long-acting reversible contraceptives (LARCs), consisting of intrauterine devices (IUDs) and implants, have a proven record of high effectiveness and high user satisfaction,^1^^-^^3^ and they are not dependent on user adherence.^4^^,^^5^ Their reversibility also makes them suitable for a vast number of women who have not completed their families.^2^ However, despite the many advantages of LARCs, contraceptive implants, which are among the most effective LARCs,^3^ make up a very small proportion of the world’s contraceptive use.^6^ In Nigeria, only 0.3% of women use implants.^7^ Among the 11% of Nigerian women who reported use of any modern method of contraception, injectables, oral contraceptives, or male condoms were most common.^7^

A study that examined 27 years of international data shows that modern contraceptive use increases with the rising number of methods available to a population.^8^ In Nigeria, most family planning clinics offer a limited method mix, namely oral contraceptive pills, injectables, and condoms. With 16% of women in Nigeria having unmet need for family planning,^7^ providing access to a variety of contraceptive methods may increase the contraceptive prevalence rate (CPR). Access to LARCs is limited in various settings due to several factors such as women’s inadequate knowledge of LARCs, lack of LARC commodities in health facilities, and inadequately skilled health care providers to render the services.^9^

Modern contraceptive use increases with the rising number of methods available to a population.

Of these barriers, the dearth of skilled providers capable of providing LARCs has been shown to be one of the most difficult to address.^10^ Evidence suggests that in settings with an insufficient number of skilled health care providers, task shifting of certain services—that is, delegating tasks to less specialized health workers—has yielded positive results.^11^^-^^14^ For example, in Ethiopia, health extension workers were trained to provide implants, and implants were more available and within the reach of many women as a result. The project was estimated to have averted 978 maternal deaths over its 3-year duration.^11^

Task shifting provision of contraceptive implants to lower-level health workers may improve access to and use of the method.

Shifting provision of contraceptive implants to lower-cadre providers such as community health extension workers (CHEWs) may be an option in addressing some of the pressing reproductive health needs in Nigeria. The purpose of this article is to describe results of an operations research study that assessed the feasibility of task shifting provision of contraceptive implants to CHEWs in 2 states of northern Nigeria and to examine facilitating factors and challenges to CHEWs providing implant insertions.

## INTERVENTION DESCRIPTION

The Targeted States High Impact Project (TSHIP), funded by the United States Agency for International Development (USAID), and the State Primary Health Care Development Agency (SPHCDA) implemented a pilot intervention to increase access to and use of contraceptive implants among rural community members in Nigeria’s Bauchi and Sokoto states. The intervention aimed to reduce unmet need for family planning in the pilot areas, improve method mix at health facilities, increase couple-years of protection (CYP), and ultimately increase the CPR in the 2 states. The intervention focused on strengthening the capacity of CHEWs to provide implants through training and supportive supervision and to document the services provided. The intervention also sought to improve commodity security and logistics systems, create demand, improve access to quality implant services, and strengthen the referral system.

### Training of CHEWs

CHEWs in Nigeria complete 2 to 3 years of formal health-related training depending on their education level at enrollment.^15^ They are full-time salaried employees in their health facilities. CHEWs were introduced primarily for community-based services (80% of their time) and some clinic-based services (20% of their time). However, as a result of acute shortages of nurses, midwives, and physicians in health facilities, the situation has been reversed with CHEWs spending 60% to 80% of their time in health facilities to provide services. For family planning services, CHEWs provide condoms, oral pills, injectables (in sites where injectables training has occurred), and emergency contraception.^15^

Family planning master trainers from the SPHCDA, with technical support from TSHIP’s family planning technical advisors, conducted a 3-week training for CHEWs from Sokoto state and a 2-week training for CHEWs in Bauchi state (the latter was shortened due to a health workers’ strike). The training aimed to provide the CHEWs with skills in administration of contraceptive implants—both Implanon (1-rod implant) and Jadelle (2-rod implant). Experienced nurses and midwives served as master trainers. Given the relatively lower level of education of the CHEWs compared with physicians, nurses, and midwives, it was important to assure that their knowledge, skills, and attitudes with respect to implant service delivery was optimal. The training for CHEWs was organized into three 7-day phases:

1. Modular teaching/learning sessions
2. Practicum sessions on implant insertions and removals on arm models
3. Supervised insertions on actual clients

In the 2-week course in Bauchi, both the didactic sessions and practice sessions on anatomic models were completed in the first week, while trainees practiced insertions on clients in the clinic during the second week. Modules focused on insertion and removal of implants and infection prevention using the competency-based approach. Other modules on interpersonal communication, balanced counseling strategy techniques, commodity logistic management system (CLMS), and using registers for recordkeeping were also essential training elements. The modules were tailored to adult learners and used participatory facilitation techniques. (See supplementary material for training materials.)

**Figure:**
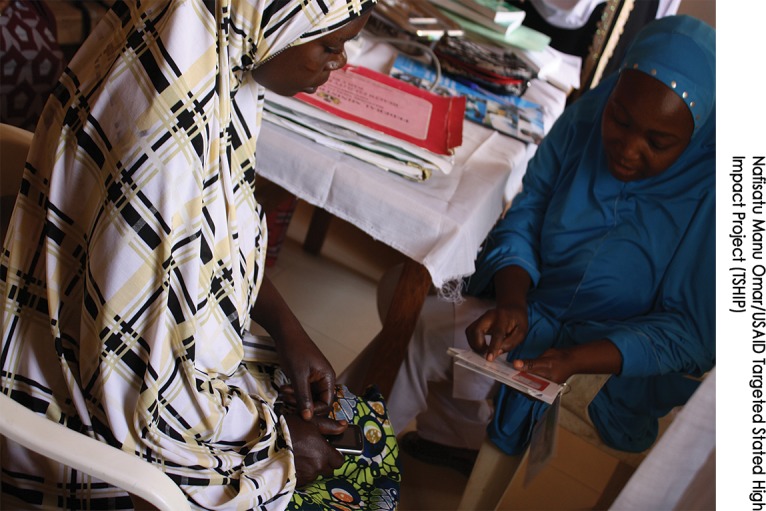
A family planning client from Bauchi state receives counseling on Jadelle implants by a Community Health Extension Worker (CHEW). Observation of counseling sessions confirmed that CHEWs provided accurate and complete information.

In the practicum sessions that followed, participants acquired implant insertion and removal skills under supervision. The learner’s guide developed for the training was available as a supportive reference. The master trainers observed each CHEW providing at least 15 implant insertions and removals on the arm model, and performance was documented using the implant procedure checklist (see supplementary material). A written pre- and post-test assessed the knowledge and skills of the CHEWs and provided insight into their level of apprehension as well as areas for improvement. After achieving competency of implant insertion and removal on the arm models, CHEWs proceeded to insert implants under supervision on actual clients in selected facilities.

### Supportive Supervision

At the end of the training sessions, the trainees and trainers jointly developed a 6-month post-training supportive supervision plan. During this period, trainers would visit trained CHEWs at health facilities to confirm they were providing services in accordance with approved standards, and the trainers would provide remedial training as needed. This period of supportive supervision also provided CHEWs the opportunity to strengthen their skills on implant insertion. We used the procedure checklists to assess skill maintenance during post-training follow-ups.

To create community awareness and increase client flow for implant uptake at various sites, trained CHEWs, in collaboration with Ward Development Committee (WDC) members and Community Based Health Volunteers (CBHVs), conducted monthly community mobilization. This helped ensure that each CHEW was provided with an adequate number of insertions under supervision. All insertions prior to certification were made in the presence of supervisors. When there were no clients during supportive supervision visits, skills were observed on anatomic models, and additional on-the-job training was provided as needed. CHEWs were certified after inserting 15 implants at their respective sites.

CHEWs were certified as competent to provide implants after inserting 15 implants.

At the end of 6 months, the trained CHEWs were linked to the state and local government area (LGA) integrated supportive supervision team for continuous service improvement. As LGA maternal and child health (MCH) coordinators, the government supervisors were previously trained on LARC methods. We provided them with a brief training to refresh their skills on implant insertion and removal as well as on supervision.

### Commodity Security and Logistics Support

The pilot intervention included ensuring adequate availability of implants (Implanon and Jadelle) in the states’ commodity stores. Monthly stock level inventories informed the timely placement of commodity orders and the delivery of implants to the 2 states. An emergency stock replenishment system that linked CHEWs with the LGA MCH coordinators was also established to promote an adequate commodity supply.

### Demand Creation Activities

WDC members, CBHVs, and CHEWs conducted sensitization activities and mobilization meetings to increase knowledge about contraceptive implants and to promote awareness and acceptance among men and women of the implant services provided by CHEWs. These activities were a part of regular family planning outreach conducted in the communities. In addition, periodic advocacy visits were made to inform key stakeholders and community gatekeepers such as religious leaders of the benefits of family planning. Demand creation activities started prior to the beginning of and continued throughout the intervention.

Implants were provided to clients free of charge. Clients were asked to pay for some of the materials needed for implant insertions.

### Provision of Implants by CHEWs Through Multiple Outlets

Trained CHEWs provided quality implant services to women through fixed clinic and mobile outreach outlets. CHEWs adhered to approved standards such as the World Health Organization’s medical eligibility criteria and national family planning/reproductive health service delivery protocols.

### Strengthening Referrals System

A 2-way referral system was established between trained CHEWs from the participating health facilities and existing primary and secondary health care facilities where midwives and doctors also provided implant services. Participating CHEWs were able to refer clients to these facilities for removals, in case of complications, and to receive feedback on the services provided and on clinical outcomes. Although CHEWs were trained in implant removal, they did not have an opportunity to practice removal skills themselves during the pilot due to low client demand for removals. Instead, we encouraged CHEWs to accompany clients requesting removal services to experienced service providers in order to gain more experience.

## METHODS

### Study Purpose and Objectives

Operations research was conducted to measure and document the feasibility and impact of the CHEWs’ task shifting pilot intervention and to report on the key lessons learned for future programming. The main objectives for the study included:

1. Assess the knowledge and skills of trained CHEWs in the provision of implants
2. Determine satisfaction of clients with services provided by CHEWs
3. Assess the extent to which the mechanisms to support CHEWs’ provision of implants functioned as intended
4. Determine facilitators and challenges encountered by CHEWs in the provision of implants

### Study Design and Sampling

We used a pre- and post-intervention study design. A random sample of 84 health facilities in Bauchi and 82 health facilities in Sokoto was selected from a sampling frame of 453 and 536 health facilities in each state, respectively. From each of the sampled facilities, 1 CHEW was randomly selected to participate in the study (166 total). To be eligible for the study, CHEWs already had to be providing family planning services in the health facility where they practiced. In addition, 1 family planning client per health facility who received services from the CHEW was randomly sampled for exit surveys. Clients were selected from women who agreed to receive implants. When no implant acceptors were available on the day of the survey, we selected respondents for exit surveys among clients who received other family planning services from the CHEW. At endline, data were collected from 151 health facilities.

### Data Collection

The study involved quantitative data collection from multiple sources, including surveys with CHEWs, client exit questionnaires, and supply checklists. We also asked CHEWs open-ended questions to examine facilitators and barriers that affected provision of implants. Additional data were gathered through a review of service statistics at health facilities and from observations of the counseling and clinical skills of CHEWs when providing implant services at health facilities.

The study protocol and all instruments were approved by the Bauchi and Sokoto State Health Research Ethics Committees and the Health Media Lab Corporation in Washington, DC. Written or verbal informed consent, depending on literacy status, was obtained from each participant.

Provider training and baseline data collection took place in September 2013 in Sokoto and in December 2013 in Bauchi. Endline data were collected 6 months later. Data were collected by nurses and midwives who received a 3-day training on the study methodology and on interviewing, observation, and documentation skills, as well as on ethics in health research.

### Data Analysis

We triangulated data from the different data sources to provide a full picture of the feasibility of using CHEWs to provide implants to women in primary health care facilities. Frequency distribution and binary analysis were conducted using Epi Info 7. Monthly analysis of service statistics was conducted for detecting trends in selected indicators.

## RESULTS

### Background Characteristics of CHEWs

The CHEWs selected for the intervention (N = 166) worked in basic and comprehensive primary health care facilities (58%), MCH units (23%), and dispensaries (19%). Some CHEWs (9%) were lost to follow-up due to their relocation to other communities or their attending college. At baseline, the median number of years working as CHEWs was 9 years (range, 1 to 35 years), and 59% of CHEWs were females. Over 70% reported they had received training in basic family planning and reproductive health in the 5 years prior to the study (72.2%, N = 162). Of the 166 CHEWs in the sample, 31% received training in family planning/reproductive health counseling, 60% in provision of oral contraceptive pills, 56% in injectable provision, 4% in insertion of IUDs, and 1% in implant insertion. Almost all CHEWs (98%) at baseline assessment reported a desire to have a refresher course in family planning. While almost all CHEWs at endline reported being able to apply the knowledge and skills acquired during implant insertion training (95%, N = 148), over 80% felt that they needed a refresher course on family planning.

Baseline data indicated that most of the health facilities where CHEWs worked (N = 166) provided pills (81%), injectables (80%), and male condoms (65%), with fewer providing female condoms (31%), and especially implants (7%) and IUDs (3%). LARCs were provided by a higher-level provider in health facilities. Family planning services were offered 5 or more days a week (88.3%, N = 137) at baseline.

### Change in CHEWs’ Implant Knowledge and Skills

The majority (94.7%, n = 143) of CHEWs received certification in implant insertion within 6 months after training. The percentage of CHEWs stating they were able to correctly insert implants without assistance increased from 6% at baseline (N = 166) to 93% at endline (N = 149) (*P*<.001). Most CHEWs who reported they were able to insert implants (n = 137) rated their skills to do so as “very good” (49%) or “good” (37%). About 14% rated their skills as “excellent.” Qualitative data analysis supports this, with most CHEWs reporting they were satisfied with their skills in implant insertion and had no difficulties or complications. Some noted that with time and with more opportunities to practice, their skills have improved.

Most CHEWs were certified in implant provision within 6 months of training.

Observation of CHEWs’ counseling skills indicated statistically significant increases (*P*<.05) from baseline to endline on 10 of the 11 observation items that addressed respectful and complete information sharing (Table 1). Endline observations confirmed that CHEWs provided accurate information on all topics related to effectiveness of contraceptive implants. The majority of CHEWs provided information on contraceptive implants (86%), explained that the method does not protect against sexually transmitted infections (STIs) including AIDS (61%), provided information about duration of protection from pregnancy (84%), gave accurate information about side effects (83%), discussed the need for the client to come back to the health facility if she experienced side effects with use (85%), reviewed the implant information card with the client (73%), provided the client with the implant information card (74%), provided the client with information on removal (83%), encouraged the client to tell friends about LARC insertion service availability at the health facility (75%), provided services in a respectful, professional manner (85%), and asked the client if she agreed to receive the implant (84%).

**Figure:**
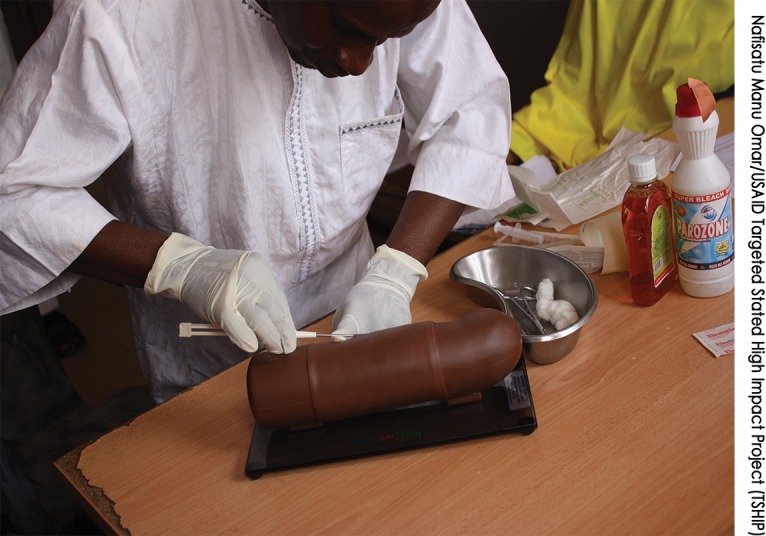
A Community Health Extension Worker (CHEW) at Dorowa Dispensary, Dambam LGA, in Bauchi state, practices inserting contraceptive implants on an arm model. After achieving competency on the arm model, CHEWs inserted implants under supervision on actual clients.

**TABLE 1:** Results of Observation of CHEWs’ Counseling Skills, Bauchi and Sokoto States of Nigeria, 2013–2014 (%)

Checklist Item	Baseline (N = 164)	Endline (N = 144)
Welcomed the client in a friendly manner	99	100
Provided information on different types of contraceptives (to new clients)	70*	96*
Asked open-ended questions	76*	88*
Encouraged client to ask questions	67*	90*
Treated client with respect	95*	100*
Saw client in private	77*	94*
Discussed a return visit	78*	89*
Asked client her concerns with any method	62*	83*
Used visual aids	31*	72*
Used client record	59*	82*
Assured client of confidentiality	52*	91*

Abbreviation: CHEW, community health extension worker.

*P<.05.

Source: Observation checklist for counseling and clinical procedures.

Clinical observation of 113 CHEWs’ doing implant insertions at endline documented high-quality service provision. Correct task performance was observed 90% of the time or more for 16 of 19 checklist items. The remaining 3 items (hand washing, asking clients to wait post-procedure, and decontamination of used items) were performed 85% of the time or more.

CHEWs performed implant tasks correctly 90% of the time or more for nearly all observation checklist items.

### Background Characteristics of Clients

We conducted exit surveys with 163 family planning clients at baseline and 150 clients at endline. Demographic characteristics of the clients were similar at both baseline and endline, with the exception of occupation and age of the youngest child. The respondents, on average, were 30 years old, had 4 children, and wanted to have 3 or 4 more children. At both assessments, over 90% of the respondents were Muslims, over 95% were married, over 75% wanted to have more children, and about 60% lacked formal education. There were more full-time housewives and fewer petty traders in the endline group than the baseline group (for housewives, 80% at endline vs. 43% at baseline, *P*<.05). On average, the age of the youngest child of the clients at endline was lower than the age of the youngest child at baseline (1.9 vs. 3.6 years, *P*<.05).

### Client Satisfaction With CHEWs’ Services

Over 95% of family planning clients at both baseline and endline reported being satisfied with the services provided on the day of the exit survey and stated that CHEWs treated them well, were friendly during the appointment, and were responsive to clients’ needs. Between baseline and endline, statistically significant increases in client satisfaction measures were documented for the percentage of respondents who felt their family planning needs were met (from 90% to 99%), who were satisfied with the amount of information provided on contraceptive methods (from 65% to 88%), and who would recommend the health facility to a friend (from 94% to 99%) (*P*<.05) (Table 2).

Virtually all clients were satisfied with the services provided by CHEWs.

**TABLE 2:** Client Satisfaction With Services Provided by CHEWs, Bauchi and Sokoto States of Nigeria, 2013–2014

Assessment	Baseline, % (N)	Endline, % (N)
Satisfied with the services provided today	99 (156)	100 (146)
Felt that needs for family planning were met	90 (159)*	99 (144)*
Thought that the right amount of information was provided on family planning method of choice	65 (153)*	88 (144)*
Thought other clients could hear what clients said	27 (148)	20 (142)
Believed the information shared with the provider would be kept confidential	86 (152)	93 (145)
Had any questions	46 (163)	56 (150)
The CHEW let clients ask the questions	90 (68)	98 (82)
The CHEW responded to questions to clients’ satisfaction	96 (53)	97 (78)
Stated that CHEW treated them well	99 (163)	99 (145)
Stated that CHEW was friendly during the appointment	97 (163)	99 (146)
Stated that CHEW was attentive and responsive to clients’ needs	99 (163)	100 (145)
Satisfied with CHEWs’ activities	98 (163)	100 (144)
Would recommend a friend to receive family planning services at this health facility	94 (156)*	99 (146)*

Abbreviation: CHEW, community health extension worker.

*P<.05.

Source: Client exit surveys.

In addition, the amount of information provided by CHEWs to clients about their chosen method increased between the 2 assessments. For example, a statistically significant increase was noted in the percentage of clients at endline who reported that CHEWs described side effects (from 79% to 95%) and told clients what to do if they had any problems (from 83% to 97%) (Table 3). There were no significant increases on 2 measures—explaining how to use the method effectively and when to return for a follow-up visit—but levels were already very high at baseline (97% and 96%, respectively).

**TABLE 3:** Information CHEWs Provided to Clients on Their Chosen Contraceptive Method, Bauchi and Sokoto States of Nigeria, 2013–2014

Information Provided by CHEWs	Baseline, % (N)	Endline, % (N)
Explained how to use the method effectively	97 (160)	99 (145)
Described possible side effects	79 (159)*	95 (145)*
Told what to do if clients have any problems	83 (157)*	97 (146)*
Explained that only condoms provide protection against STIs	54 (159)*	71 (143)*
Told how many years of protection against pregnancy the method provides	82 (158)*	97 (144)*
Told when to return for a follow-up visit	96 (158)	92 (144)

Abbreviation: CHEW, community health extension worker; STIs, sexually transmitted infections.

*P<.05.

Source: Client exit survey.

### Uptake of Implants

The proportion of health facilities that provided implants increased from 7% at baseline (N = 166) to 91% (N = 150) at endline (*P*<.001). According to service statistics, a total of 3,588 implants were inserted at 151 health facilities by endline (2,723 Implanon and 865 Jadelle insertions), or 4 implants per health facility per month. Over a period of 6 months, 10,088 CYP were generated through implant insertion.

Almost 90% of clients at endline did not pay for the services provided (89%, N = 146). Those who paid reported paying for hospital cards and disposable materials and felt those expenses were affordable.

### Performance of Structural Support Mechanisms

#### Training

Most CHEW respondents were satisfied with the training. In their responses to open-ended questions, CHEWs noted a good balance between theory and practice and a high quality of instruction, and they said they were provided with enough opportunities to practice. They also said their knowledge and skills had improved. Participants were happy to learn about the reproductive system and other contraceptive methods during the training. Also, they were thrilled to be able to practice on actual clients. Some respondents said they needed more time for practicing their skills so that “practical skills are perfected.” Knowledge based on the post-test and skills assessment based on observations did not differ significantly between participants completing the 2-week vs. the 3-week training.

#### Supply System

According to facility observations using supply checklists (N = 149), implants were available in 90% of all facilities on the day of the endline survey. Guidelines and protocols for inserting implants were available in two-thirds of the facilities (67%). Nearly all (90%) of the health facilities kept clients’ records in a secured area. Availability of supplies necessary for implant insertions improved significantly from baseline to endline (*P*<.001). Nevertheless, only about two-thirds of all surveyed health facilities at endline had the supplies necessary for implant insertions (e.g., xylocaine, sterile gloves, adhesive bandages).

Some facilities did not have all the supplies needed for implant insertion, such as sterile gloves.

According to responses to open-ended questions, CHEWs reported using daily consumption registers, national health information system monthly summary forms, and family planning review meeting summary forms. CHEWs report monthly to the MCH in the LGA and every 2 months to the CLMS review meeting. CHEWs reported receiving their supply of implants from MCH coordinators and resupply at the review meetings. If needed, they could use emergency orders, in which case supplies would be provided within 2 days. In closed-ended survey questions, only 8% of CHEWs reported stock-outs of implants in the last 6 months (N = 134), which could be resupplied in 1 week or less in over 80% of cases.

#### Supportive Supervision

Essentially all (95%; N = 145) of the CHEWs who received training on contraceptive implants at baseline also received supervisory and monitoring visits in the post-training time frame. The number of visits ranged from 1 to 8 over a 6-month period. According to CHEWs’ responses to open-ended questions, supervisors observed implant insertions and made corrections if needed, answered questions, and provided feedback on counseling. For the CHEWs who had not done any insertions, supervisors gave advice on how to increase client flow. Thus, they encouraged CHEWs to work more on community mobilization, provide more counseling, and sensitize women in the community.

The majority of CHEWs reported the supervision and feedback to be very helpful. According to their reports, visits encouraged them to perform better and provided opportunities to communicate their problems and receive support. Most CHEWs received feedback during and immediately after the supervision visit. However, a few reported not receiving any feedback. To improve supervision, CHEWs recommended more frequent supervision visits—once or twice every month—to provide feedback during and after each visit. CHEWs also recommended providing supervisors with logistics support, e.g., transportation to hard-to-reach areas.

### CHEWs’ Satisfaction With Providing Implant Services

CHEWs’ responses to open-ended questions provided information on changes in their workload due to task shifting, facilitating factors and challenges to their providing implants, and suggestions for making their work sustainable in the community. Most of CHEWs who worked on implant insertions reported there was no substantial increase in workload, although they reported having more responsibilities and duties such as filling out reporting forms. Regardless, CHEWs stated they enjoyed their service to the community. CHEWs felt privileged for receiving this opportunity, found inserting implants interesting and satisfying, and felt comfortable implementing this new task. While the task added extra work for CHEWs, they were happy to gain new experience, skills, and knowledge. Also, they felt honored that women who came for implant insertions trusted that CHEWs would do a good job. Some stated that now they are respected more by community members.

Most CHEWs indicated there was no substantial increase in workload with the addition of implant insertion responsibilities.

Among the main facilitating factors in identifying implant clients, CHEWs noted community mobilization efforts, advocacy work, and work of volunteers to increase women’s awareness on the method. Among other facilitators of increased uptake, CHEWs cited consistent availability of implants and provision of the method free of charge.

CHEWs remarked that their main challenges included low acceptance of implants by community members due to misconceptions (e.g., “implants cause infertility,” “they can’t be removed from the body,” “they can’t be found after insertion since they move inside the body”) and religious beliefs against family planning in general. Also, some women were used to receiving injectables and were reluctant to try a new contraceptive method. Fear of side effects such as headache, spotting after insertion, irregular periods, and the necessity to purchase some of the consumables were stated as other challenges.

In terms of additional support needed to administer implants, CHEWs expressed a strong desire for training and retraining. They also suggested training more staff in implant insertion to increase the number of available service providers.

CHEWs offered suggestions for making their work sustainable in the community. Most suggestions related to increasing demand for implants in the community via community mobilization (Box). Other suggestions included providing high-quality services, ensuring regular supply of free implants and all materials for implant insertion, and offering clients small gifts or incentives. Family planning clients at endline (N = 150) recommended the following ways to encourage more women in the community to receive family planning services: increasing women’s awareness on family planning service availability (85%), receiving encouragement from religious leaders (45%), and having CHEWs communicate with husbands (41%).

**BOX. Ways to Increase Demand for Implants via Community Mobilization**

CHEWs offered the following suggestions as ways to increase demand among communities for implants:

- Provide continuous health education and distribute more information, education, and communication (IEC) materials, especially in the local (Hausa) language.
- Use more volunteers to carry out home-to-home visits to increase awareness and address fears and misconceptions.
- Conduct advocacy among community and religious leaders; involve Ward Development Committee members and husbands at a larger scale.
- Use all opportunities to create demand such as antenatal care visits, immunization visits, health talks, and community gatherings.

## DISCUSSION

Shifting provision of contraceptive implants to CHEWs in northern Nigeria was successful—clinical observations showed that CHEWs consistently followed the standard protocols and consequently delivered high-quality services, confirming previous findings from other countries.^11^ In addition, although the CHEWs reported a slight workload increase as a result of the task shifting intervention, they were satisfied with their performance and results of their work. CHEWs rated their skills in implant insertions as high and felt confident in performing the procedure. We also found that CHEWs’ counseling skills improved over time and that they retained these skills throughout the duration of the study. Furthermore, the CHEWs’ reported increased job satisfaction, which was directly associated with their added responsibility of implant insertion. Their satisfaction is a welcome development in overall health worker motivation and could extend to other aspects of the CHEWs’ work.

CHEWs in northern Nigeria were able to provide high-quality implant insertion services.

**Figure:**
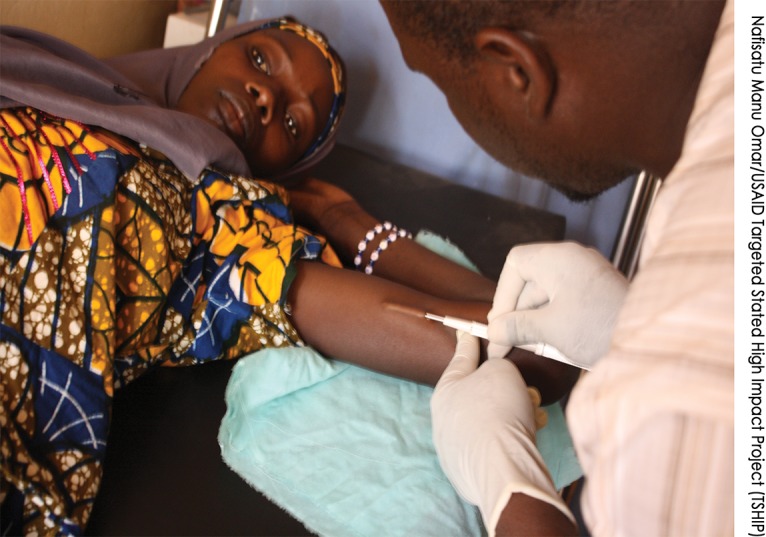
A male Community Health Extension Worker (CHEW) from Muzuwa Dispensary, Dambam LGA, in Bauchi state, inserts a contraceptive implant. Over 40% of the CHEWs in the pilot task shifting study were men, revealing the acceptance of male providers by women to deliver implant services.

This study provides evidence in support of the potential to replicate task shifting of contraceptive implants to CHEWs in Nigeria. It also provides evidence in support of a recently adopted national policy statement by Nigeria’s Council of Health in October 2014, wherein task shifting was adopted as a national policy.^16^ Although the number of implant insertions per health facility per month was relatively low in this pilot study, we think this underscores the importance of working with communities to address misconceptions about implants and to increase demand for implant services in addition to scaling-up training of CHEWs to provide the service. Initially, as client demand for implant services begins to grow, training and using “dedicated providers” to offer implant insertions and removals might be the most practical solution to ensure providers have an opportunity to practice and maintain the skills they learn during training. These providers can then become mentors to their (trained) colleagues to offer similar services in the future.

Community outreach is needed to address misconceptions and to generate demand for implant services.

Such an expansion of trained providers with increased client demand will accelerate the trend of declining total fertility rate (TFR) in Sokoto state, which dropped from 8.7 in 2008 to 7.0 in 2013.^7^^,^^18^ Similarly, this intervention could contribute to a decline in Bauchi state’s TFR, which remained stagnant at 8 during the same period. Evidence of increased demand for services will be a strong advocacy tool to health or district managers for assigning additional staff to the family planning clinics. With increasing popularity of LARCs,^17^ the study suggests that providing contraceptive implant services via task shifting might be a good way to meet family planning needs.

To our knowledge, this is the first study that trained CHEWs in insertion of both 1-rod and 2-rod implants. While there were no differences in the availability of Implanon and Jadelle for the CHEWs and clients, Implanon composed three-quarters of all insertions. This could be explained by preferences of clients to space their births 3 years apart (effective duration of Implanon) rather than 5 years, the effective duration of Jadelle (although Jadelle, as with any long-acting method, can be removed before the effective duration of the method expires). Also, because Implanon contains only 1 rod, it is quicker to insert than the 2-rod Jadelle implant. For Jadelle, providers need to learn to insert the first rod, then turn the trocar 20 degrees to insert the second rod. We noticed that for less than a quarter of CHEWs, this skill took longer to master than that of Implanon insertion. However, as soon as competency was achieved, the only difference between Implanon and Jadelle insertion was the duration of insertion, which is usually only a difference of a few seconds.

The crucial role of supportive supervision in maintaining quality assurance was also noted. The number of supervision visits varied depending on individual needs of CHEWs until competency was achieved. The project’s integration of supportive supervision into the government-run supervision system is likely to increase the sustainability of such a support system for CHEWs. However, the CHEWs’ preferred frequency of 3 to 6 supervisory support visits per quarter exceeds the project’s current routine supportive supervision guidelines. It will be important to monitor and establish the minimum level of supervisory contact required for the optimal functioning of the LARC task shifting endeavor; this could be the basis of further operations research.

The study collected data about policies and procedures at health facilities. Client satisfaction with CHEW services was high, indicating that staff were friendly and able to answer clients’ questions. Since over 40% of the CHEWs in the study were men, the high level of client satisfaction suggests the acceptance of male providers by women to deliver contraceptive implant services in the context of northern Nigeria. Guidelines on implant insertions were available in most health facilities, and adequate reporting systems were in place. However, several areas for improvement need to be addressed, including improving availability of information, education, and communication (IEC) materials, providing more frequent supportive supervision, and providing immediate feedback after each supervision visit.

The study also looked at issues of availability of implants, including stock-outs, and materials at health facilities. While implants were available in most health facilities, supply of consumables such as sterile gloves, adhesive bandages, and antiseptics in both states was insufficient, a situation that needs to be addressed urgently to prevent infection.

Several studies have identified country-specific barriers for uptake of family planning methods overall^19^^,^^20^ and of some particular methods such as IUDs and tubal ligation.^21^ Our study contributes to the existing research by identifying barriers to uptake of implant services. These include women’s fears of implants causing infertility, of the side effects of implants, or that implants can’t be removed and could disappear in the body. Targeted health communication messages need to be developed and disseminated to community members to correct these misconceptions and to encourage use of implants and other contraceptive methods.

### Strengths and Limitations

This study has several limitations. First, although the results of the study may be used to inform interventions in Bauchi and Sokoto states, we need to exercise caution generalizing findings and recommendations to other states of Nigeria. In addition, the selected CHEWs in these 2 states had prior family planning training conducted by TSHIP and other organizations. Therefore, CHEWs in other states might need to be trained in family planning prior to being trained on implant insertion. Also, in the surveys with CHEWs and clients, we relied on self-reported data, which may be subject to social desirability bias. We tried to minimize biases by training data collectors on proper survey techniques and ensuring respondents of data confidentiality. Attrition could be a threat to internal validity in our study; however, characteristics of those 9% of CHEWs who were lost to follow-up did not differ from those who stayed in the study. We did not assess the costs of the provision of implants by CHEWs. Future research should conduct cost-effectiveness analysis to answer questions regarding affordability and relative effectiveness of this intervention versus alternative options to promote and increase use of contraceptive implant services. Trainees were not able to practice implant removal skills on clients due to low demand for these services. Currently, health facilities are linked to general hospitals where CHEWs are able to remove implants under the supervision of experienced service providers. We plan to retrain CHEWs on removals as the client load for removal increases. Additional research is needed to compare off-site group-based training with on-site, shorter yet more frequent training for CHEWs. The benefit of the latter is that health care workers are not taken away from the job posts for a long time period. Finally, although we presented CHEWs’ insights regarding keeping the intervention sustainable in the future, this topic was beyond the scope of our study. We believe the approved national task shifting policy that allows CHEWs to be trained to provide implants and increasing interest in LARCs facilitated the task sharing intervention. Future investigations should rigorously examine factors that support scale-up and sustainability of the intervention.

Among the study’s strengths is that the findings are based on results of surveys and clinical observations as well as a review of service statistics. In addition to survey questions with set response options, we asked CHEWs open-ended questions to get respondents’ insight on facilitating factors and barriers for providing services. We are confident in our findings because triangulation of results from different data sources indicates that the findings reinforce each other.

## LESSONS LEARNED

The following are key lessons learned from our pilot intervention in Nigeria that can be used to improve access to implants in underserved communities.

1. CHEWs can provide quality contraceptive implant services with adequate support including continual supportive supervision and regular refresher training. Continual supervision is necessary to ensure high-quality services are provided. Regular feedback during and after each supervisory visit motivates CHEWs to improve their performance. We also recommend refresher training on counseling and clinical skills to improve quality of services.
2. The 2-week training duration was adequate to ensure competently trained CHEWs. We did not find differences in knowledge and skills between participants in the 2-week vs. 3-week trainings, suggesting that 2 weeks is adequate if the training is competency-based and focused on implant insertion and removal.
3. Implant insertions with 5 clients were sufficient to achieve competency and confidence. At the beginning of the pilot, we set an arbitrary goal of 15 insertions per CHEW to gain competency. Experience, however, has shown that when trainees master skills repeatedly on anatomic models in the classroom, they need a much lower number of insertions on clients to achieve competency and confidence. Based on our experience, trainees who achieved competency on anatomic models during classroom simulation practice with appropriate procedure checklists also achieved competency on clients after 4–5 procedures. This is similar to the findings from an IUD competency-based training in Thailand where 70% of learners were judged to be competent after 2 insertions with clients and 100% were competent after 6 insertions.^22^
4. A combination of interventions addressing the demand and supply sides of implant provision would increase uptake and use of services. Future programs should make efforts to:
  - **Ensure availability of implants and other supplies:** Policies to ensure availability of implants and other materials necessary for implant insertion should be in place to prevent stock-outs.
  - **Create demand:** Demand for implant services in communities should be created prior to introducing the services, and community mobilization work should be ongoing. Misconceptions and fears regarding implants and family planning overall are common. While some CHEWs reported an increased uptake in implants, a few did not insert any implants because they had no clients. As part of the pilot program, we conducted community mobilization activities in all communities where sampled CHEWs worked. However, we did not promote implants exclusively; instead, we promoted use of family planning methods, including implants. A more focused and intense effort toward demand creation that is based on volunteerism and informed choice could have yielded a higher uptake of implants among women in the communities.
  - **Develop and disseminate targeted messages to community members using information materials in a local language:** Key messages for community mobilization activities need to be developed and disseminated to promote implant uptake. Distribution of information materials in a local language would facilitate the acceptance of implants by community members.

Most CHEWs achieved competency in implant insertion after 5 insertions with client.

## CONCLUSION

With adequate training that included supportive supervision, CHEWs provided high-quality implant insertions. Despite their increased workload due to new reporting requirements and administration of implants, CHEWs enjoyed learning new skills and applying them in their daily work. With training of more CHEWs in implant insertion and additional community outreach to generate demand for services, uptake of LARC methods in Nigeria may increase. Investing in supportive supervision and use of a standards-based supervisory checklist will help ensure sustainability of the task shifting intervention.

## Notes

**Peer Reviewed**

## References

[1] Curry DW Rattan J Huang S Noznesky E. Delivering high-quality family planning services in crisis-affected settings II: results. Glob Health Sci Pract. 2015;3(1):25–33. 10.9745/GHSP-D-14-00112. 25745118 PMC4356273

[2] Jacobstein R Stanley H. Contraceptive implants: providing better choice to meet growing family planning demand. Glob Health Sci Pract. 2013;1(1):11–17. 10.9745/GHSP-D-12-00003. 25276512 PMC4168562

[3] Trussell J. Contraceptive failure in the United States. Contraception. 2011;83(5):397–404. 10.1016/j.contraception.2011.01.021. 21477680 PMC3638209

[4] Blumenthal PD Voedisch A Gemzell-Danielsson K. Strategies to prevent unintended pregnancy: increasing use of long-acting reversible contraception. Hum Reprod Update. 2011;17(1):121–137. 10.1093/humupd/dmq026. 20634208

[5] Wickstrom J Jacobstein R. Contraceptive security: incomplete without long-acting and permanent methods of family planning. Stud Fam Plann. 2011;42(4):291–298. 10.1111/j.1728-4465.2011.00292.x. 22292248

[6] Ross J Keesbury J Hardee K. Trends in the contraceptive method mix in low- and middle-income countries: analysis using a new “average deviation” measure. Glob Health Sci Pract. 2015;3(1):34–55. 10.9745/GHSP-D-14-00199. 25745119 PMC4356274

[7] National Population Commission (NPC) [Nigeria]; ICF International Nigeria demographic and health survey 2013. Abuja (Nigeria): NPC; 2014 Co-published by ICF International. Available from: http://dhsprogram.com/publications/publication-FR293-DHS-Final-Reports.cfm

[8] Ross J Stover J. Use of modern contraception increases when more methods become available: analysis of evidence from 1982-2009. Glob Health Sci Pract. 2013;1(2):203–212. 10.9745/GHSP-D-13-00010. 25276533 PMC4168565

[9] Secura GM Allsworth JE Madden T Mullersman JL Peipert JF. The Contraceptive CHOICE Project: reducing barriers to long-acting reversible contraception. Am J Obstet Gynecol. 2010;203(2):115.e1–e7. 10.1016/j.ajog.2010.04.017. 20541171 PMC2910826

[10] World Health Organization (WHO) From evidence to policy: expanding access to family planning. Optimizing the health workforce for effective family planning services. Geneva: WHO; 2012 Available from: http://apps.who.int/iris/bitstream/10665/75164/1/WHO_RHR_HRP_12.19_eng.pdf

[11] Asnake M Cole C Oliveras E Tilahun Y Scale-up of task-shifting for community-based provision of Implanon: 2009-2011 technical summary. Watertown (MA): Pathfinder International; 2011 Available from: http://www.pathfinder.org/publications-tools/Scale-Up-of-Task-Shifting-for-Community-Based-Provision-of-Implanon-Technical-Summary-2009-2011.html

[12] Hoke TH Wheeler SB Lynd K Green MS Razafindravony BH Rasamihajamanana E Community-based provision of injectable contraceptives in Madagascar: ‘task shifting’ to expand access to injectable contraceptives. Health Policy Plan. 2012;27(1):52–59. 10.1093/heapol/czr003. 21257652

[13] Lehmann U Van Damme W Barten F Sanders D. Task shifting: the answer to the human resources crisis in Africa? Hum Resour Health. 2009;7(1):49. 10.1186/1478-4491-7-49. 19545398 PMC2705665

[14] Malarcher S Meirik O Lebetkin E Shah I Spieler J Stanback J. Provision of DMPA by community health workers: what the evidence shows. Contraception. 2011;83(6):495–503. 10.1016/j.contraception.2010.08.013. 21570545

[15] Advancing Partners & Communities Country profile: Nigeria community health programs. Arlington (VA): Advancing Partners & Communities; 2014 Available from: https://www.advancingpartners.org/sites/default/files/landscape/countries/profiles/country_profile_nigeria.pdf

[16] Federal Ministry of Health (MOH) [Nigeria] Task-shifting and task-sharing policy for essential health care services in Nigeria. Abuja (Nigeria): MOH; 2014 Available from: http://advancefamilyplanning.org/sites/default/files/resources/Nigeria%20taskshifting%20policy-Aug2014%20REVISEDCLEAN%20_Approved%20October%202014.pdf

[17] Stunning popularity of LARCs with good access and quality: a major opportunity to meet family planning needs. Glob Health Sci Pract. 2015;3(1):12–13. 10.9745/GHSP-D-15-00044. 25745116 PMC4356271

[18] National Population Commission (NPC) [Nigeria]; ICF International Nigeria demographic and health survey 2008. Abuja (Nigeria): NPC; 2009. Co-published by ICF Macro Available from: http://dhsprogram.com/publications/publication-fr222-dhs-final-reports.cfm

[19] Aninyei I Onyesom I Ukuhor H Uzuegbu U Ofili M Anyanwu E. Knowledge attitude to modern family planning methods in Abraka communities, Delta State, Nigeria. East Afr J Public Health. 2008;5(1):10–12. 18669116 10.4314/eajph.v5i1.38970

[20] Mairiga AG Kullima AA Bako B Kolo MA Sociocultural factors influencing decision-making related to fertility among the Kanuri tribe of north-eastern Nigeria. Afr Prim Health Care Fam Med. 2010;2(1):1–4. 10.4102/phcfm.v2i1.94.

[21] Chigbu B Onwere S Aluka C Kamanu C Okoro O Feyi-Waboso P. Contraceptive choices of women in rural Southeastern Nigeria. Niger J Clin Pract. 2010;13(2):195–199. 20499755

[22] Jhpiego Training skills for health care providers: reference manual. 3rd ed. Baltimore (MD): Jhpiego; 2010 Available from: http://reprolineplus.org/resources/training-skills-health-care-providers-third-edition-reference-manual

